# Prevalence of Germline Mutations in Genes Engaged in DNA Damage Repair by Homologous Recombination in Patients with Triple-Negative and Hereditary Non-Triple-Negative Breast Cancers

**DOI:** 10.1371/journal.pone.0130393

**Published:** 2015-06-17

**Authors:** Pawel Domagala, Anna Jakubowska, Katarzyna Jaworska-Bieniek, Katarzyna Kaczmarek, Katarzyna Durda, Agnieszka Kurlapska, Cezary Cybulski, Jan Lubinski

**Affiliations:** 1 Department of Pathology, Pomeranian Medical University, Szczecin, Poland; 2 Department of Genetics and Pathology, International Hereditary Cancer Center, Pomeranian Medical University, Szczecin, Poland; CNR, ITALY

## Abstract

**Purpose:**

This study sought to assess the prevalence of common germline mutations in several genes engaged in the repair of DNA double-strand break by homologous recombination in patients with triple-negative breast cancers and hereditary non-triple-negative breast cancers. Tumors deficient in this type of DNA damage repair are known to be especially sensitive to DNA cross-linking agents (e.g., platinum drugs) and to poly(ADP-ribose) polymerase (PARP) inhibitors.

**Methods:**

Genetic testing was performed for 36 common germline mutations in genes engaged in the repair of DNA by homologous recombination, i.e., *BRCA1*, *BRCA2*, *CHEK2*, *NBN*, *ATM*, *PALB2*, *BARD1*, and *RAD51D*, in 202 consecutive patients with triple-negative breast cancers and hereditary non-triple-negative breast cancers.

**Results:**

Thirty five (22.2%) of 158 patients in the triple-negative group carried mutations in genes involved in DNA repair by homologous recombination, while 10 (22.7%) of the 44 patients in the hereditary non-triple-negative group carried such mutations. Mutations in *BRCA1* were most frequent in patients with triple-negative breast cancer (18.4%), and mutations in *CHEK2* were most frequent in patients with hereditary non-triple-negative breast cancers (15.9%). In addition, in the triple-negative group, mutations in *CHEK2*, *NBN*, and *ATM* (3.8% combined) were found, while mutations in *BRCA1*, *NBN*, and *PALB2* (6.8% combined) were identified in the hereditary non-triple-negative group.

**Conclusions:**

Identifying mutations in genes engaged in DNA damage repair by homologous recombination other than *BRCA1/2* can substantially increase the proportion of patients with triple-negative breast cancer and hereditary non-triple-negative breast cancer who may be eligible for therapy using PARP inhibitors and platinum drugs.

## Introduction

Triple-negative breast cancer (TNBC), i.e., breast cancer characterized by no immunohistochemical expression of the estrogen receptor (ER) or progesterone receptor (PR) and the absence of human epidermal growth factor receptor 2 (HER-2) overexpression, accounts for 15–20% of breast cancer cases [1]. Patients with TNBC are characterized by a high risk of relapse, poor prognosis and insensitivity to anti-estrogen and anti-HER2 targeted therapies [2]. Thus, this subset of breast cancers is mainly responsible for the difficulties encountered during efforts to improve the survival of patients with breast cancer. TNBCs constitute approximately 80% of *BRCA1*-associated breast cancers [3]. However, *BRCA1/2* mutations have only been found in a subset of patients with TNBC [4]. Women with a strong family history of breast cancer or breast and ovarian cancers are at increased risk of this disease compared with the general population. Approximately 30% of all hereditary breast cancer patients and the majority of breast and ovarian cancer patients harbor germline mutations in the *BRCA1/2* genes [5].

The cytotoxic effect of radiotherapy and commonly used chemotherapeutic drugs is a result of DNA damage, which can be limited by DNA repair mechanisms within tumor cells. Several alternative DNA repair pathways are known. Of these, homologous recombination repairs DNA double-strand break with high fidelity [6]. Functionally competent *BRCA1* and *BRCA2* genes are essential for DNA damage repair by homologous recombination. Tumors in patients carrying germline mutations in these genes exhibit homologous recombination deficiency and are especially sensitive to DNA cross-linking agents (e.g., platinum drugs) and poly(ADP-ribose) polymerase (PARP) inhibitors [7]. However, DNA damage repair by homologous recombination is a complex, multistep process that involves not only *BRCA1* and *BRCA2* but also other genes. DNA damage repair involves the recruitment and coordinated interactions of specific proteins (DNA damage sensors, mediators, transducers and effector proteins) to maintain the integrity of the genome. PALB2 is a critical mediator of homologous recombination in human cells; therefore, PALB2-deficient cells are sensitive to PARP inhibitors [8]. A protein product of the *NBN* gene (nibrin) is a component of the MRE11/RAD50/NBN (MRN) protein complex, which is involved in repair of DNA damage by homologous recombination and non-homologous end joining [9]. *CHEK2*, which is associated with an intermediate risk for breast cancer and several other cancers [10], codes for a protein kinase that is downstream of the ATM and MRN complex in the DNA damage signaling cascade and transduces signals in response to DNA damage to regulators of apoptosis and the cell cycle. ATM is required for accurate DNA double-strand break repair to prevent the accumulation of unrepaired double-strand break and genomic instability [11]. BARD1 plays a role in the response to DNA damage as a stoichiometric binding partner of BRCA1. Each BRCA1–BARD1 super complex is responsible for executing distinct elements of BRCA1-dependent damage response activity [12]. RAD51D is involved in DNA damage repair through homologous recombination and the founder mutation in this gene was recently reported in patients with a family history of breast and ovarian cancer [13].

Studies in cell lines have indicated that a deficiency of repair proteins is associated with the ineffective repair of DNA damage by homologous recombination and renders tumor cells sensitive to PARP inhibitors through a synthetic lethality mechanism [14]. Hence, it is reasonable to expect that patients with germline mutations in genes involved in DNA damage repair by homologous recombination may be candidates for treatment using PARP inhibitors and platinum drugs. However, little is known regarding germline mutations in the genes engaged in DNA damage repair by homologous recombination except for *BRCA1/2* in patients with TNBC. Furthermore, virtually nothing is known of mutations in these repair genes in patients with hereditary non-triple-negative breast cancers (Hn-TNBCs). Finding mutations in these genes could increase the proportion of patients with TNBC who may be eligible for treatment with PARP inhibitors and platinum therapy and, at the same time, reduce the number of TNBC patients for whom no targeted therapy is available. Identification of such mutations may also single out those patients with Hn-TNBCs who may be eligible for such therapies. Therefore, there is a need for comprehensive analysis of the prevalence of mutations in genes involved in DNA repair in patients with TNBCs and Hn-TNBCs.

The aim of this study was to assess the prevalence of common germline mutations in several genes that are components of the homologous recombination pathway of DNA damage repair, i.e., *BRCA1*, *BRCA2*, *CHEK2*, *NBN*, *ATM*, *PALB2*, *BARD1*, and *RAD51D*, among unselected cohorts of patients with TNBCs and Hn-TNBCs. Mutations in these genes are known to be associated with ineffective repair of DNA damage by homologous recombination.

## Materials and Methods

### Ethics

Informed written consent was obtained from each patient, and this study was approved by the Ethics Committee at the Pomeranian Medical University (decision No. BN-001/33/04).

### Patients

From a cohort of 1,255 consecutive breast cancer patients described previously [15] we studied a group of 165 consecutive women with TNBC unselected for family history and a group of 46 consecutive patients with Hn-TNBC. Nine cases were excluded because DNA could not be adequately amplified for all studied variants after repeated attempts, leaving 202 eligible cases (158 TNBCs and 44 Hn-TNBCs). Patients with non-triple-negative breast cancer and a history of breast cancer or breast and ovarian cancer at any age of diagnosis in at least two relatives, one of whom was the first-degree relative to the other two or the second- degree relative through a male, were classified as having Hn-TNBC [16]. Based on the triple-negative immunophenotype of breast cancer and the family history two groups were distinguished: patients with TNBC unselected for family history and patients with Hn-TNBC. The clinicopathologic patient characteristics are shown in Table 1. Pathology and immunohistochemistry (ER, PR, HER-2) review was conducted as described previously [15]. Only first primary invasive breast carcinomas were included. A detailed family history concerning cancers in relatives was available for 97% (1220/1255) of the initial cohort. A family history was taken either by constructing a family tree or completing a standardized questionnaire. All first- and second-degree relatives diagnosed with breast cancer and their ages at diagnosis were recorded.

**Table 1:** Clinicopathological characteristics of the study groups.

Characteristics	Triple-negative n (%)	Hereditary non-triple-negative n (%)
Age at diagnosis (years)		
Range	23–85	31–80
Mean	55.5	57.5
Mean number of breast cancers in families	1.37	3.00
Mean number of ovarian cancers in families	0.11	0.21
Tumor grade		
G1	0	8 (18.2)
G2	17 (10.8)	24 (54.5)
G3	141 (89.2)	12 (27.3)
Lymph node status		
N0	112 (70.9)	25 (56.8)
N1	46 (29.1)	19 (43.2)
Tumor size		
≤ 2 cm	80 (51.3)	31 (70.5)
> 2 cm	76 (48.7)	13 (29.5)
Histopathological type		
Ductal	97 (61.3)	32 (72.8)
Medullary	14 (8.9)	0
Atypical medullary	27 (17.1)	0
Metaplastic	6 (3.8)	0
Lobular	3 (1.9)	6 (13.6)
Other	11 (7.0)	6 (13.6)
ER		
Negative	158 (100)	5 (11.4)
Positive	0	39 (88.6)
PR		
Negative	158 (100)	6 (13.6)
Positive	0	38 (86.4)
HER-2		
Negative	158 (100)	6 (13.6)
Positive	0	38 (86.4)

### Selecting mutations

We chose 36 mutations in eight genes involved in DNA double-strand break repair by homologous recombination (Table 2). These mutations are known to be associated with ineffective repair of DNA damage by homologous recombination. Because approximately 80% of patients carrying the *BRCA1* mutation have TNBC [3], a high prevalence of *BRCA1* mutations can be expected in this group; hence, we studied all of the pathogenic variants in *BRCA1* that have been described in the Polish population (references in Szwiec et al. [17]). Mutations in *BRCA2* are rare in the Polish population (0.4% in early onset breast cancers), and because there are no founder mutations, we selected five *BRCA2* mutations that were previously reported in four or more unrelated Polish women [17]. Mutations in *CHEK2*, *NBN*, *PALB2* (c.509_510delGA), and *ATM* were reported as recurrent in the Polish population [18–24]. Four founder mutations of *CHEK2* were previously studied in this group, and the incidence in the TNBC subgroup was published elsewhere [15]. In addition to epidemiologic data [25,26], recent studies based on DNA damage assays [9,27] showed that missense variants in *CHEK2* c.470T>C (p.I157T) and *NBN* c.511A>G (p.I171V) are pathogenic; therefore, we included these variants in this study. Furthermore, two new recurrent mutations recently discovered in *PALB2* (c.1592delT) and *RAD51D* (c.576+1G>A) in the Finnish population [13,28] were also tested. To date, these mutations have not been reported in the Polish population. Additionally, we included rare pathogenic variants in *BARD1* (c.1690C>T, c.1315-2A>G) that have recently been described in the Polish population [29].

**Table 2:** List of tested variants.

Gene	Variants^1^
*BRCA1*	c.5266dupC, c.181T>G, c.4035delA, c.3700_3704delGTAAA, c.68_69delAG, c.5251C>T, c.3756_3759delGTCT, c.1687C>T, c.3936C>T, c.5030_5033delCTAA, c.675delT, c.2563C>T, c.2866_2870delTCTCA, c.3286C>T, c.5346G>A, c.190T>C, c.4484+1G>A, c.5406+5G>A, c.2872_2876delTTCAG
*BRCA2*	c.658_659delGT, c.3847_3848delGT, c.5239_5240insT, c.5946delT, c.7910_7914delCCTTT
*CHEK2*	c.1100delC, c.444+1G>A, del5395 (exon 10-11del), c.470T>C
*NBN*	c.657_661delACAAA, c.511A>G
*PALB2*	c.509_510delGA, c.1592delT
*ATM*	c.5932G>T
*BARD1*	c.1690C>T, c.1315-2A>G
*RAD51D*	c.576+1G>A

^1^Mutation type according to the HGVS nomenclature

### Genetic testing

Each patient was approached for genetic testing after diagnosis. Genomic DNA was prepared from 5–10 ml of peripheral blood leukocytes. Nine founder mutations in *BRCA1*, *CHEK2*, *NBN*, and *ATM* were genotyped as described previously [18,20,24]. In brief, *BRCA1* mutations (c.5266dupC and c.4035delA) and *NBN* (c.657_661delACAAA), were detected using allele-specific oligonucleotide PCR, and c.181T>G was detected using restriction fragment length polymorphism PCR. The *CHEK2* del5395 mutation was detected by a multiplex PCR reaction. The c.444+1G>A and c.470T>C variants in *CHEK2* were detected using restriction fragment length polymorphism PCR analysis, and the c.1100delC mutation was analyzed using allele-specific oligonucleotide PCR. Remaining selected mutations were genotyped using TaqMan assays (Life Technologies, Foster City, CA) on LightCycler 480 II instrument (Roche, Germany). DNA testing results indicating the occurrence of mutations were confirmed by Sanger sequencing.

### Statistics

Fisher’s exact test was used for categorical variables to determine differences between groups. Logistic regression was used to assess impact of age at diagnosis on the probability of carrying mutation in genes involved in DNA damage repair by homologous recombination. Statistical analyses were performed using GraphPad Prism 6 software (San Diego, CA) and for logistic regression in R statistical environment v. 3.2. For all statistical analyses, a *P* value < 0.05 was considered significant.

## Results

The results are summarized in Tables 3 and 4 and detailed raw data are supplied in S1 Table. In the group of patients with TNBC, a germline *BRCA1* mutation was identified in 29 of 158 (18.4%) patients, a *CHEK2* mutation in 3 (1.9%), an *NBN* mutation in 4 (2.5%), and an *ATM* mutation in 1 (0.6%) patient. Altogether there were 35 of 158 (22.2%) patients with mutations in genes involved in DNA damage repair by homologous recombination (18.4% in *BRCA1* and 3.8% in the other genes). When patients with a mutation in *BRCA1* were excluded, 6/129 (4.7%) TNBC patients with mutations in the other selected genes remained.

**Table 3:** Prevalence of germline mutations in genes tested in patients with triple-negative breast cancer and hereditary non-triple-negative breast cancer.

Gene	Triple-negative n = 158	%	Hereditary non-triple-negative n = 44	%
*BRCA1*	27	17.2	1	2.3
*CHEK2*	2	1.3	5	11.3
*NBN*	3	1.9	1	2.3
*PALB2*	0	0	1	2.3
*ATM*	1	0.6	0	0
*BRCA1/CHEK2* ^1^	1	0.6	0	0
*BRCA1/NBN* ^2^	1	0.6	0	0
*CHEK2/NBN* ^3^	0	0	2	4.5
**All**	35	22.2	10	22.7

Four patients carried two different mutations:

^1^ *BRCA1*-c.3700_3704delGTAAA/*CHEK2*-c.470T>C;

^*2*^ *BRCA1*-c.3700_3704delGTAAA/*NBN*-c.511A>G;

^3^ *CHEK2*-c.444+1G>A/*NBN*-c.511A>G and *CHEK2*-c.470T>C/*NBN*-c.657_661delACAAA.

**Table 4:** Prevalence of germline mutations in genes tested in patients with early onset (≤50) triple-negative and hereditary non-triple-negative breast cancer.

Gene	Triple-negative n = 54	%	Hereditary non-triple-negative n = 12	%
*BRCA1*	16	29.5	1	8.3
*CHEK2*	1	1.9	1	8.3
*PALB2*	0	0	1	8.3
*BRCA1/CHEK2*	1	1.9	0	0
*BRCA1/NBN*	1	1.9	0	0
*CHEK2/NBN*	0	0	1	8.3
**All**	19	35.2	4	33.3

Among the 44 patients with Hn-TNBC, we identified 10 patients (22.7%) with mutations in genes involved in DNA damage repair by homologous recombination. There were 7 (15.9%) patients with mutations in *CHEK2*, one with a mutation in *PALB2*, one with a mutation in *NBN*, and one with a mutation in *BRCA1*. Mutations in genes other than *CHEK2* were found in 6.8% of cases. All of the tumors except for 5 in the Hn-TNBC group were ER-positive breast cancers, and in this ER-positive group, there were 25.6% (10/39) of patients with mutations in genes involved in DNA repair by homologous recombination.

We did not find mutations in *BRCA2*, *BARD1*, or *RAD51D*. We found one patient with the *PALB2* c.509_510delGA mutation but none with c.1592delT. Two double heterozygous breast cancer cases were identified in the TNBC group (one patient was *BRCA1/CHEK2* and the other was *BRCA1/NBN*). Two subjects in the Hn-TNBC group had germline mutations in both *CHEK2* and *NBN* (Table 3).

In patients with early onset (≤50 years) breast cancer the percentage of patients with mutations in genes involved in DNA damage repair by homologous recombination increased to 35.2% and 33.3% in the TNBC and Hn-TNBC groups, respectively (Table 4). In the TNBC group, mutations in *BRCA1* increased from 18.4% in patients unselected for age to 33.3% in patients with early onset breast cancer. In the TNBC group a subset of early onset patients had significantly higher mutation rate than others (35.2% vs. 15.4%, *P* = 0.008) whereas a comparison of the mutation rates for early onset patients vs. others within the Hn-TNBC group did not show statistical significance (33.3% vs. 18.8%, *P* = 0.42).

**Fig 1:**
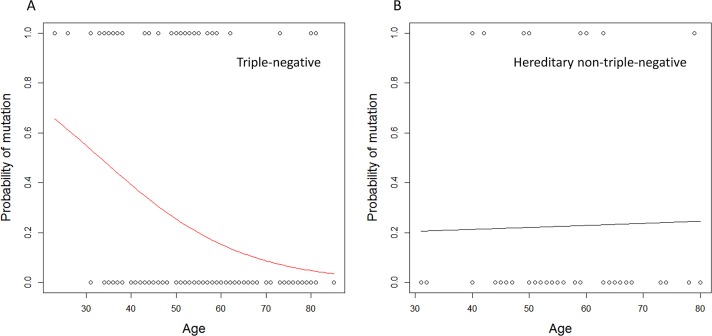
Probability of carrying a mutation in genes involved in DNA repair by homologous recombination depending on age at diagnosis of triple-negative breast cancer (A) and hereditary non-triple-negative breast cancer (B). Fig. 1 was generated using the generalized linear model (glm) function in R environment. See the online supplementary R script (S1 Text).

The probability of carrying a mutation in genes involved in DNA repair by homologous recombination depending on the age at diagnosis of TNBC is shown in Fig 1. The probability of being a mutation carrier was 51% when the diagnosis of TNBC was made by 32 years of age but only 15% at 60 years of age. Among patients in the Hn-TNBC group, the probability of carrying a mutation was nearly constant (21% at age 32 and 23% at age 60).

The overall prevalence of mutations in genes involved in the homologous recombination pathway was similar in the TNBC and Hn-TNBC groups (22.2% vs. 22.7%, *P* = 1.00); however, the types of mutations were different in patients with TNBC and Hn-TNBC. Mutations in *BRCA1* were most frequent in patients with TNBC (18.4% vs. 2.3%, *P* = 0.007), whereas mutations in *CHEK2* were most frequent in patients with Hn-TNBC (15.9% vs. 1.9%, *P* = 0.001).

## Discussion

In this report we provide new information on the prevalence of common germline mutations in genes involved in DNA double-strand break repair by homologous recombination in patients with TNBC and Hn-TNBC. We identified germline mutations in several genes involved in the homologous recombination pathway in addition to the well-known and well-characterized *BRCA1/2*. The results show that a significant number of patients with TNBC and Hn-TNBC harbor mutations in other genes involved in homologous recombination, which may indicate that such patients are candidates for an extended range of therapies that induce specific forms of DNA damage or that inhibit PARP.

Recently, it has become apparent that germline mutations in genes that are part of the homologous recombination machinery are not only associated with the development of breast cancer and various human cancers but may also influence the sensitivity of breast cancers to therapy because defects in the homologous recombination pathway are associated with hypersensitivity to PARP inhibitors and other chemotherapeutic agents [30,31]. Cells expressing clinically relevant *BRCA1* mutations are deficient in DNA damage repair by homologous recombination [32]. Genetic testing for mutations in *BRCA1* has proven to be useful in identifying patients most likely to benefit from DNA cross-linking agents (e.g., platinum drugs [33]) and targeted therapies utilizing a synthetic lethality concept [7]. The reported prevalence rates of germline *BRCA1* mutations in patients with TNBC range from 10% to 42% [4,34,35]. A recent meta-analysis [34] showed that 22% of selected high-risk breast cancer patients with TNBC were carriers of *BRCA1* mutations. However, various criteria were used to define high-risk groups in the 12 studies included in this meta-analysis (575 patients with TNBC accrued from 12 cohorts with fewer than 100 patients each). In our unselected cohort of patients with TNBC, *BRCA1* mutations were found in 18.4% of patients. Other studies have reported prevalence values of 11.1% [4], 15% [36], and 15.6% [37] in unselected patients with TNBC. *BRCA1* mutation prevalence depends on the age of the patient at diagnosis of TNBC, and prevalence is higher in younger patients. In patients with early onset (≤50 years old) TNBC, the prevalence was 27.6% in Sharma’s report [4] and 33.3% in our study. By contrast, in unselected women with early onset breast cancer *BRCA1* mutations were found in 7.1% of patients [17]. Hence, there is strong evidence that one of the major underlying defects associated with homologous recombination deficiency in patients with TNBC is mutation in *BRCA1*. In Poland three *BRCA1* mutations (c.5266dupC, c.4035delA, c.181T>G) accounted for 82–91% of the mutation positive families [38–40]. In this study we detected pathogenic variants in *BRCA1* that have been described in the Polish population (references in Szwiec et al [17]) including five founder mutations (c.5266dupC, c.4035delA, c.181T>G, c.3700_3704delGTAAA, c.5251C>T) and other rare mutations (c.5030_5033delCTAA, c.1687C>T, c.3936C>T). These mutations in *BRCA1* have also been described in other populations e.g., in the Ashkenazi Jewish, Austrian, Slovenian, German, Czech, Finnish, and Greek populations [41]. In the TNBC and Hn-TNBC groups we detected germline mutations in *BRCA1* in 18.4% and 2.3% of patients respectively. The frequency of *BRCA1* mutations in population of Poland is estimated at about 0.3–0.4% [42]. The most frequent *BRCA1* mutation (c.5266dupC) was detected in 0.17% of population controls [42].

The majority of all pathogenic mutations in *BRCA1* are frameshift (e.g., c.5266dupC, c.4035delA, c.3700_3704delGTAAA, c.68_69delAG, c.5030_5033delCTAA) or nonsense (e.g., c.5251C>T, c.1687C>T, c.3936C>T), and they yield a truncated protein product [43]. However, some deleterious *BRCA1* mutations are missense changes that occur in key conserved protein domains such as the ring finger domain (e.g., c.181T>G) and the BRCA1 C terminal (BRCT) domain [44]. Functional studies of the c.181T>G mutation show that it results in inactivation of *BRCA*1 E3 ligase activity and is defective in homologous recombination [44,45].

We found no mutations in the *BRCA2* gene in our groups of patients, which is consistent with the rare incidence of *BRCA2* mutations in the Polish population [17]. Furthermore, mutations in *BRCA2* are associated with a luminal immunophenotype [46]; therefore, they will be very rare in an unselected group of patients with a high prevalence of TNBC.

Nevertheless, a number of genes other than *BRCA1/2* encode proteins involved in DNA double-strand break repair by homologous recombination; mutations in these genes could increase the likelihood of responsiveness to PARP inhibitors (or other inhibitors) and platinum compounds [47]. However, the prevalence of germline mutations in genes other than *BRCA1/2* in patients with TNBCs and Hn-TNBCs is largely unknown. Here, we report that other common germline mutations in genes involved in DNA repair by homologous recombination, i.e., in *CHEK2*, *NBN*, *ATM* and *PALB2* were detected in these two groups of patients in addition to mutations in *BRCA1*. In the TNBC group germline mutations in *NBN*, *CHEK2* and *ATM* genes were detected in 2.5%, 1.9% and 0.6% of patients respectively. In Hn-TNBC group germline mutations in *CHEK2*, *NBN*, and *PALB2* genes were found in 15.9%, 6.8% and 2.3% of patients respectively. For comparison, in Poland the *NBN* c.657_661delACAAA and c.511A>G mutations combined were detected in 0.8% [18,21,48,49], *PALB2* mutation c.509_510delGA was detected in 0.08% [19], and *ATM* mutation c.5932G>T was reported in 0.05% of population controls [20]. *CHEK2* truncating mutations (c.1100delC, c.444+1G>A, del5395) were detected in 1% and the missense mutation c.470T>C was found in 4.8% population controls in Poland [15,50].

The *PALB2* germline deletion c.509_510delGA creates a premature stop codon and leads to a shortened PALB2 protein, which is devoid of the C-terminal domain that appears to be necessary for BRCA2/PALB2 complex formation [51] and homologous recombination repair [19]. Thus, monoallelic *PALB2* loss-of-function mutations result in a truncated PALB2 protein that retains little BRCA2-binding capacity and results in deficient homologous recombination [28]. The *NBN*-c.511A>G variant reduces the DNA damage repair activity of NBN, elevates chromosomal instability and increases the risk of breast cancer [9]. The *NBN* c.657_661delACAAA mutation results in a frameshift and a truncated protein with loss of expression, possibly leading to sensitivity to PARP inhibitors [14,52,53]. Cells with a c.5932G>T mutation in *ATM* exhibit loss of ATM protein expression [54], and ATM protein deficiency sensitizes cells to PARP inhibition therapy [14,55]. Because BRCA1 phosphorylation by CHEK2 is required for homologous recombination pathway activity [56], loss of *CHEK2* expression (via a truncating mutation) or its inability to phosphorylate BRCA1 (c.470T>C mutation) may result in synthetic lethality in the presence of PARP inhibitors [57]. Cells with the c.470T>C variant of *CHEK2* exhibit no response to DNA damage [27], although a synergistic effect with deficiency of CHEK2 in tumor cells may also be induced by drugs that target microtubules (e.g., taxanes) [57]. However, the efficacy of neoadjuvant therapy was shown to be particularly poor in *CHEK2* carriers receiving anthracyclines without taxanes [58]. Homologous recombination pathway proteins are known to harbor significant numbers of pathogenic missense substitutions, and it is believed that the vast majority of genetic risk attributable to *BRCA1*, *BRCA2* and *PALB2* is due to protein-truncating variants. In contrast, *ATM* and *CHEK2* belong to a group of genes in which half or more of their attributable genetic risk is caused by rare missense substitutions [59]. Recently, a growing recognition of the role of rare missense substitutions in breast cancer susceptibility has been emphasized [59]. For example, the fraction of breast cancer risk attributable to rare missense substitutions in three susceptibility genes, *TP53*, *ATM* and *CHEK2*, is estimated to be as high as the fraction of risk attributable to protein-truncating variants.

Thus the mutations we detected in patients with TNBC and Hn-TNBC contribute to a deficient homologous recombination response [8,9,14,27,32,47,53,55,57]. Therefore, it seems likely that testing patients with TNBC and Hn-TNBC for mutations in genes involved in homologous recombination may improve the identification of women who could benefit from therapy utilizing DNA cross-linking agents (e.g., platinum drugs) and PARP inhibitors or inhibitors of other components of the homologous recombination pathway. Furthermore, we found double heterozygous breast cancer mutations in two patients with TNBC (one patient was *BRCA1/NBN*) and two patients with Hn-TNBC. Although the number of such patients was small, this subset of patients may exhibit significant homologous recombination deficiency and be particularly sensitive to PARP inhibitors and platinum therapy. Indeed, triple-negative HCC1395 cells deficient in both nibrin and BRCA1 have been reported to be particularly sensitive to PARP1 inhibition [60].

For the first time we have distinguished a group of patients with Hn-TNBC based on clinicopathological criteria. Although the overall prevalence of mutations in genes involved in homologous recombination was similar in patients with TNBC and Hn-TNBC, mutations in *BRCA1* were most frequent in the former group, while mutations in *CHEK2* were most frequent in the latter group. Patients with Hn-TNBC were also more heterogeneous than patients with TNBC in terms of their clinical and pathological characteristics. The probability of carrying mutations in genes engaged in DNA damage repair by homologous recombination at 32 years of age reached approximately 51% in patients with TNBC, and this percentage is likely to increase as new, rarer mutations are revealed by next-generation sequencing.

The goal of modern therapy of breast cancer is a precise, personalized and targeted approach that provides the patient with the best available treatment for her particular unique cancer. One important aspect of this approach is making available existing therapeutic options that may only be appropriate for small but definitively characterized subsets of breast cancers. In the current report, we show that testing patients with TNBC and Hn-TNBC for germline mutations in genes involved in the homologous recombination pathway can identify patients who may have specific therapeutic options. To avoid excessive toxicity, each patient should expect to receive treatment that is best tailored to her genetic/molecular status. Identifying mutations in genes associated with homologous recombination (other than *BRCA1/2*) may increase the likelihood of responses to PARP inhibitors as a single agent and may also allow reduced dosing, thereby minimizing the risk of the serious hematologic toxicities associated with platinum treatment.

## Conclusions

We identified germline mutations in *BRCA1* and several other genes involved in DNA double-strand break repair by homologous recombination in patients with TNBC and Hn-TNBC. These patients could potentially benefit from therapy utilizing PARP inhibitors and DNA cross-linking agents (e.g., platinum derivatives), although they would not become candidates for such therapies if they were tested only for mutations in *BRCA1/2*. Our data support the usefulness of detection of carriers of mutations in genes involved in homologous recombination for appropriate therapy selection for hereditary breast cancer patients [61] depending on mutation frequencies and the presence of specific founder or recurrent mutations in the population. From the point of view of DNA repair by homologous recombination triple-negative breast cancers were characterized by preponderance of *BRCA1* mutations whereas hereditary non-triple-negative breast cancers by preponderance of *CHEK2* mutations over mutations in other genes involved in this type of DNA repair tested in our report. Our results indicate that a *BRCA1/2*-centered perspective may ignore the significance of additional, non-negligible mutations in genes engaged in DNA damage repair by homologous recombination that may influence therapy outcome. Furthermore, identifying breast cancer patients with homologous recombination deficiencies associated with germline mutations other than *BRCA1/2* mutations seems to be necessary for the design of therapies based on synthetic lethality and for the interpretation of results of clinical trials aimed at evaluating the response to PARP inhibitors (or PARP inhibitors combined with chemotherapy regimens), not only in TNBC patients but also in those with Hn-TNBC.

## Supporting Information

S1 Table Age of patients and germline mutations identified in the study groups. (XLSX)

S1 Text R script used to generate Fig 1. (PDF)
