# Supplementary material for: Prevalence of Germline Mutations in Genes Engaged in DNA Damage Repair by Homologous Recombination in Patients with Triple-Negative and Hereditary Non-Triple-Negative Breast Cancers
Source: PLoS One. 2015 Jun 17;10(6):e0130393. doi: 10.1371/journal.pone.0130393 (PMC4471155; doi:10.1371/journal.pone.0130393)
Supplement: S1 Text — (PDF) [file pone.0130393.s002.pdf]

Triple-negative:

```
dat <- structure(list(Mutation = c(0,0,0,0,0,0,0,0,0,0,0,0,0,0,
0,0,0,0,0,0,0,0,0,0,0,0,0,0,0,0,0,0,0,0,0,0,0,0,0,0,0,0,0,0,
0,0,0,0,0,0,0,0,0,0,0,0,0,0,0,0,0,0,0,0,0,0,0,0,0,0,0,0,0,0,
0,0,0,0,0,0,0,0,0,0,0,0,0,0,0,0,0,0,0,0,0,0,0,0,0,0,0,0,0,0,
0,0,0,0,0,0,0,0,0,0,0,0,0,0,0,0,0,1,1,1,1,1,1,1,1,1,1,1,1,1,1,
1,1,1,1,1,1,1,1,1,1,1,1,1,1,1,1,1,1,1,1),
Age = c(31,34,35,35,36,37,38,40,40,41,42,43,43,43,43,44,
44,45,45,45,46,46,46,47,47,47,47,48,48,50,50,50,50,50,50,
51,51,51,51,52,52,53,53,53,53,53,54,54,54,54,54,55,55,55,
55,55,56,56,56,56,56,56,57,57,57,57,58,59,59,59,59,59,59,
60,60,60,60,60,61,61,61,61,62,62,62,62,63,64,64,65,65,65,
66,66,67,67,67,68,68,68,68,70,70,70,70,71,73,73,74,75,76,
76,76,77,78,79,80,80,80,81,81,81,85,23,26,31,33,34,35,36,
37,38,43,44,44,44,44,44,46,49,49,50,51,51,52,52,53,53,54,
55,55,57,58,59,62,73,80,81)),
Names = c("Mutation", "Age"),
class = "data.frame", row.names = c(NA, 158))
age.glm <- glm(Mutation ~ Age, data=dat, family=binomial)
plot(type="p", dat$Age, dat$Mutation, xlab="Age",
ylab="Probability of mutation", cex.lab=1.5)
curve(predict(age.glm, data.frame(Age=x), type="resp"),
add=TRUE, col="red")
```

Hereditary non-triple-negative:

```
dat <- structure(list(Mutation = c(0,0,0,0,0,0,0,0,0,0,0,0,0,
0,0,0,0,0,0,0,0,0,0,0,0,0,0,0,0,0,0,0,1,1,1,1,1,1,1,1,1,1,1),
Age = c(78,45,64,56,74,55,32,53,50,46,31,67,55,67,68,65,
59,52,56,80,56,59,54,51,58,73,66,63,44,56,52,80,47,40,42,
60,79,79,40,60,49,63,59,50)),
Names = c("Mutation", "Age"),
class = "data.frame", row.names = c(NA, 44))
age.glm <- glm(Mutation ~ Age, data=dat, family=binomial)
plot(type="p", dat$Age, dat$Mutation, xlab="Age",
ylab="Probability of mutation", cex.lab=1.5)
curve(predict(age.glm, data.frame(Age=x), type="resp"),
add=TRUE, col="black")
```
